# DDA‐imaging with structural identification of lipid molecules on an Orbitrap Velos Pro mass spectrometer

**DOI:** 10.1002/jms.4882

**Published:** 2022-09-02

**Authors:** Saleh M. Khalil, Richard R. Sprenger, Martin Hermansson, Christer S. Ejsing

**Affiliations:** ^1^ Department of Biochemistry and Molecular Biology, VILLUM Center for Bioanalytical Sciences University of Southern Denmark Odense Denmark; ^2^ Cell Biology and Biophysics Unit European Molecular Biology Laboratory Heidelberg Germany

**Keywords:** lipid identification, lipidomics, MALDI‐imaging mass spectrometry, mouse brain

## Abstract

Matrix‐assisted laser desorption/ionization‐mass spectrometry imaging (MALDI‐MSI) is a useful technique for visualizing the spatial distribution of lipid molecules in tissues. Nevertheless, the use of MSI to investigate local lipid metabolic hallmarks has until recently been hampered by a lack of adequate technology that supports confident lipid identification. This limitation was recently mitigated by the development of DDA‐imaging technology where high‐resolution MSI is combined with parallel acquisition of lipid tandem MS^2^ spectra on a hybrid ion trap‐Orbitrap Elite mass spectrometer featuring a resolving power of 240,000 and a scan time of 1 s. Here, we report the key tenets related to successful transfer of the DDA‐imaging technology onto an Orbitrap Velos Pro instrument featuring a resolving power of 120,000 and a scan time of 2 s. Through meticulous performance assessments and method optimization, we tuned the DDA‐imaging method to be able to confidently identify 73 molecular lipid species in mouse brain sections and demonstrate that the performance of the technology is comparable with DDA‐imaging on the Orbitrap Elite. Altogether, our work shows that DDA‐imaging on the Orbitrap Velos Pro instrument can serve as a robust workhorse for lipid imaging in routine applications.

## 1 INTRODUCTION

Matrix‐assisted laser desorption mass spectrometry imaging (MALDI‐MSI) is a useful analytical technique for imaging the spatial distribution of a wide range of biomolecules, including lipids.^1^, ^2^ Hundreds of putative lipid molecular ions in tissue sections can be visualized routinely and easily with a spatial resolution of 40 μm^3^ and with extra effort these can be mapped to a spatial resolution of 1.4 μm.^4^ MALDI‐MSI technology has so far been used for lipid imaging in diverse biological systems, including individual cell culture cells,^5^ tissues from animals,^6^ microbes,^7^ plants,^8^ parasites,^9^ and insects.^10^ Nevertheless, a key challenge in lipid imaging is deciphering whether detected molecular ions are genuine lipid molecules or false‐positive annotations related to other molecules with various adduct ions (e.g., H^+^, Na^+^, ^37^K^+^, and ^41^K^+^), isotopologues, in‐source fragmentation as well as chemical noise.

One way to mitigate false‐positive lipid annotations is to use Fourier transform (FT) ion cyclotron resonance or Orbitrap mass spectrometry.^11^, ^12^ High‐resolution FTMS^1^ analysis affords accurate mapping of putative lipid molecular ions based solely on accurate *m/z* values and identification at the lipid species‐level with notation of the total number of carbon atoms, double bonds and hydroxyl groups in *all* hydrocarbon chains. However, the high‐resolution FTMS^1^ analysis alone can still yield a high degree of false‐positive lipid annotations,^13^ representing “lipid molecules” that are unlikely based on known lipid metabolic pathways and that cannot be confirmed by lipid analysis of homogenized tissue extracts.

Another way to reduce false‐positive lipid annotations is to combine the high‐resolution FTMS^1^‐based imaging with data‐dependent (DDA) acquisition of lipid tandem MS^2^ spectra at a single pixel‐level.^14^ In doing so, it becomes possible to identify lipids at the molecular species‐level with notation of the number of carbon atoms, double bonds, and hydroxyl groups in individual hydrocarbon chains, and importantly, confirm whether a particular FTMS^1^‐based image stem from a genuine lipid molecule or not. This DDA‐imaging technology was recently established on a hybrid ion trap (IT)‐Orbitrap Elite mass spectrometer, which due to its hardware design allows parallel recording of FTMS^1^ and ITMS^2^ spectra with a scan time around 1 s per pixel.^14^ Analysis of the FTMS^1^ and ITMS^2^ data by the lipidomic software ALEX^123^ ^15^ allowed high‐confidence identification of 113 molecular lipid species in tissue sections of rat cerebellum. Notably, transferring the DDA‐imaging technology to other instruments comes with an overhead of assessing and benchmarking the performance in reference to fixed, hardware‐related specifications (e.g., number of detectors, resolving power) as well as tunable acquisition method‐specific parameters (e.g., DDA settings).

Here, we outline the implementation and validation of DDA‐imaging technology on a hybrid IT‐Orbitrap Velos Pro, which inherently features an inferior resolving power and scan time as compared with the Orbitrap Elite. Nevertheless, through systematic assessment of hardware‐specific specifications, optimization of acquisition method‐specific parameters, and benchmarking, we show that the performance of DDA‐imaging is comparable with that of the Orbitrap Elite. Overall, our method validation shows that DDA‐imaging on the Orbitrap Velos Pro can serve as a robust workhorse for lipid imaging in routine applications.

## 2 MATERIALS AND METHODS

### 2.1 Chemicals

Methanol, 2‐propanol, and water were purchased from Biosolve BV (Valkenswaard, Netherlands). Chloroform was from Rathburn Chemicals (Walkerburn, UK). Ammonium formate and norharmane were from Sigma‐Aldrich (Buchs, Switzerland). All solvents and chemicals were HPLC grade. Synthetic lipid standards were purchased from Avanti Polar Lipids (Alabaster, USA).

### 2.2 Mouse brain section preparation

Animal experiments were conducted in accordance with German law (in congruence with 86/609/EEC) for the use of laboratory animals and approved by the local animal welfare committee at the Johannes Gutenberg University Mainz. Male C57BL/6 wild‐type mice (8 weeks old) were euthanized by an overdose of ketamine by intraperitoneal injection. Subsequently, the mice were perfused intracardially with cold 155 mM ammonium acetate, and the brains were quickly removed, snap‐frozen in liquid nitrogen and stored at −80°C until sectioned, as previously described.^16^ For MALDI imaging experiments, frozen brain tissue was cut into 15 μm thick sections in a cryostat (CM 3050 S; Leica Microsystems, Nussloch, Germany), placed on a glass slide, and stored at −80°C until further processing.

### 2.3 Matrix deposition for MALDI‐MSI

Tissue sections were put in a desiccator for 20 min to minimize condensation of atmospheric water on their surfaces. For MSI in positive and negative ion mode, norharmane (7 mg/ml) in chloroform:methanol (7:3, v/v) was applied with an iMatrixSpray (Tardo, Switzerland). Spray conditions were as follows: height, 60 mm; line distance, 1 mm; speed, 180 mm/s; density, 1 μl/cm^2^; cycles, 15; delay, 0 s; pressure, 1.6 bar.

### 2.4 DDA‐imaging

DDA‐imaging experiments were performed on an Orbitrap Velos Pro mass spectrometer (Thermo Fisher Scientific, Bremen, Germany) equipped with an elevated‐pressure MALDI source incorporating a dual‐ion funnel interface (Spectroglyph LLC, Kennewick, WA, USA), as described.^3^, ^14^ The integrated Q‐switched frequency‐tripled Nd:YLF laser (349 nm) was used at a repetition rate of 1 kHz and pulse energy of ~1.2–1.4 μJ. The laser was focused and controlled to a spot size diameter of ~15 μm, as confirmed by measuring the burn pattern in matrix deposited on a glass slide by light microscopy (data not shown). The sample stage of the MALDI source and the high‐pressure ion funnel was maintained at 7.5–7.8 Torr. The low‐pressure ion funnel was maintained at 1.6–1.7 Torr. Applied radio frequency voltages to the high‐ and low‐pressure ion funnels were set to 590 kHz, 210 V_0‐peak_ and 890 kHz, 80 V_0‐peak_, respectively. Ejection of MALDI‐generated ions into the ion funnels was accomplished by an electric field  gradient of ~100 V/cm between the sample holder and the first ion funnel electrode.

For DDA‐imaging experiments, the MALDI stage step size was set to 20 μm (horizontal) × 40 μm (vertical) with a speed of 4 mm/s. Parallel full‐scan FTMS^1^ (Orbitrap) and ITMS^2^ (ion trap) scans at neighboring 20 μm positions were accomplished using an instrument method consisting of two repeating scan events. The first was a 100,000 resolution FTMS^1^ scan from *m/z* 180–2000. The second was a DDA‐ITMS^2^ scan. As such, one FTMS^1^ and one ITMS^2^ scan were acquired from each 40 × 40 μm sampling region, which is equivalent to one pixel in the acquired MSI data. Both FTMS^1^ and DDA‐ITMS^2^ scans were completed at the same time; such that the parallel ITMS^2^ acquisitions do not add any extra time to the overall runtime of the DDA‐imaging experiment. To improve the mass accuracy of the FTMS^1^ analysis, online lock‐mass function was used. Reference masses in negative and positive ion mode were norharmane‐derived ions with *m/z* 333.11457 and *m/z* 335.12912 in negative and positive ion mode, respectively.^17^ Generation of *m/z* images was done using a custom script written in MATLAB (Mathworks, Natick, MA, USA)^14^ as well as Image Insight software (Spectroglyph LLC, Kennewick, WA, USA). Every *m/z* image is normalized to the total ion count and displayed using the same color scale.

### 2.5 Lipid nomenclature

Lipids reported at the “species‐level” are denoted by their lipid class abbreviations, followed by the total number of carbon atoms, double bonds and hydroxyl groups in all hydrocarbon chains. For example, “SM 36:1;2” denotes a SM lipid with a total of 36 carbon atoms, 1 double bond, and 2 hydroxyl groups in its two hydrocarbon chains.^18^ Lipids reported at the “molecular species‐level” are denoted by their lipid class abbreviations, followed by the number of carbon atoms, double bonds, and hydroxyl groups in each hydrocarbon chain. For example, “PE 18:0‐22:6” indicates a PE lipid containing an 18:0 and a 22:6 fatty acyl (FA) chain.

### 2.6 Lipid identification

Identification of lipid molecules was done using the ALEX^123^, as previously described.^14^ Briefly, lipid precursor ions detected by FTMS^1^ were identified using a *m/z* tolerance of ±0.0045 Da, corrected for potential ^13^C isotope interference, required to have a relative detection frequency greater than 0.5 (equivalent to being detected in 50% of all DDA‐imaging data files of identical polarity) and annotated at the “species‐level.” Lipid fragment ions detected by ITMS^2^ were identified using a *m/z* tolerance of ±0.2 Da, required to have a relative detection frequency greater than 0.50 (equivalent to being detected in 50% of all DDA‐imaging data files of identical polarity) and annotated as “molecular lipid species‐specific fragments” (MLF) or “lipid class‐specific fragments” (LCF).^19^ For high‐confidence identification of molecular lipid species identified by detection of MLFs, the following criteria were set: (i) The asymmetric molecular lipid species must be detected by at least two complementary pairs of MLFs (except for protonated PE O‐ species that do not release abundant complementary MLFs); (ii) the molecular lipid species must have an ALEX^123^ score >0.5 (calculated as the number of detected MLFs relative to the total number of MLFs available in the ALEX^123^ database) or an ALEX^123^ score ≤0.5 but with detection of >2 MLFs (with the exception that protonated PE O‐ species could be detected by at least 2 MLFs); and (iii) confirmation by detection of the corresponding lipid molecule at the species‐level by full‐scan FTMS.

### 2.7 Imaging of tissue sections by optical microscopy

Optical microscopy was performed on a Nikon Eclipse Ti microscope (Nikon, Japan). Tissue sections were stained with hematoxylin and eosin (H&E), and images were acquired at 10× magnification.

## 3 RESULTS AND DISCUSSION

### 3.1 Transfer of DDA‐imaging technology

**FIGURE 1:**
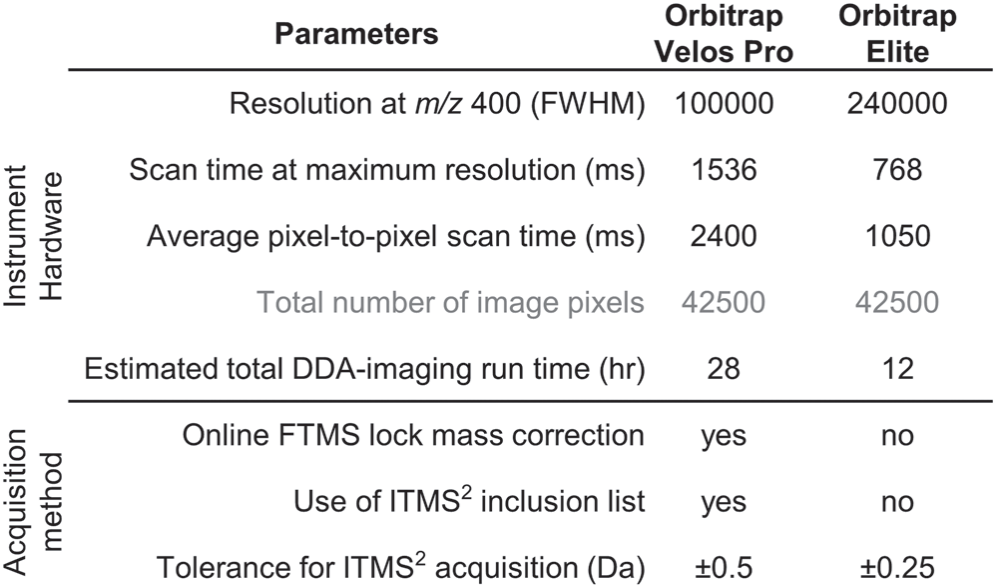
Key parameters that can influence the performance of DDA‐imaging technology. The parameters can be grouped into those related to the hardware of the mass spectrometers, which cannot be modified, and those that are related to data acquisition, which to some extent can be modified.

While transferring the DDA‐imaging technology from the Orbitrap Elite instrument to an Orbitrap Velos Pro machine, we identified several analytical parameters that warranted further investigation and adaptation to optimize performance. Overall, these parameters covered details related to the hardware of the instrumentation and settings of the DDA‐imaging method for parallel acquisition of FTMS^1^ and ITMS^2^ spectra (Figure 1).

**FIGURE 2:**
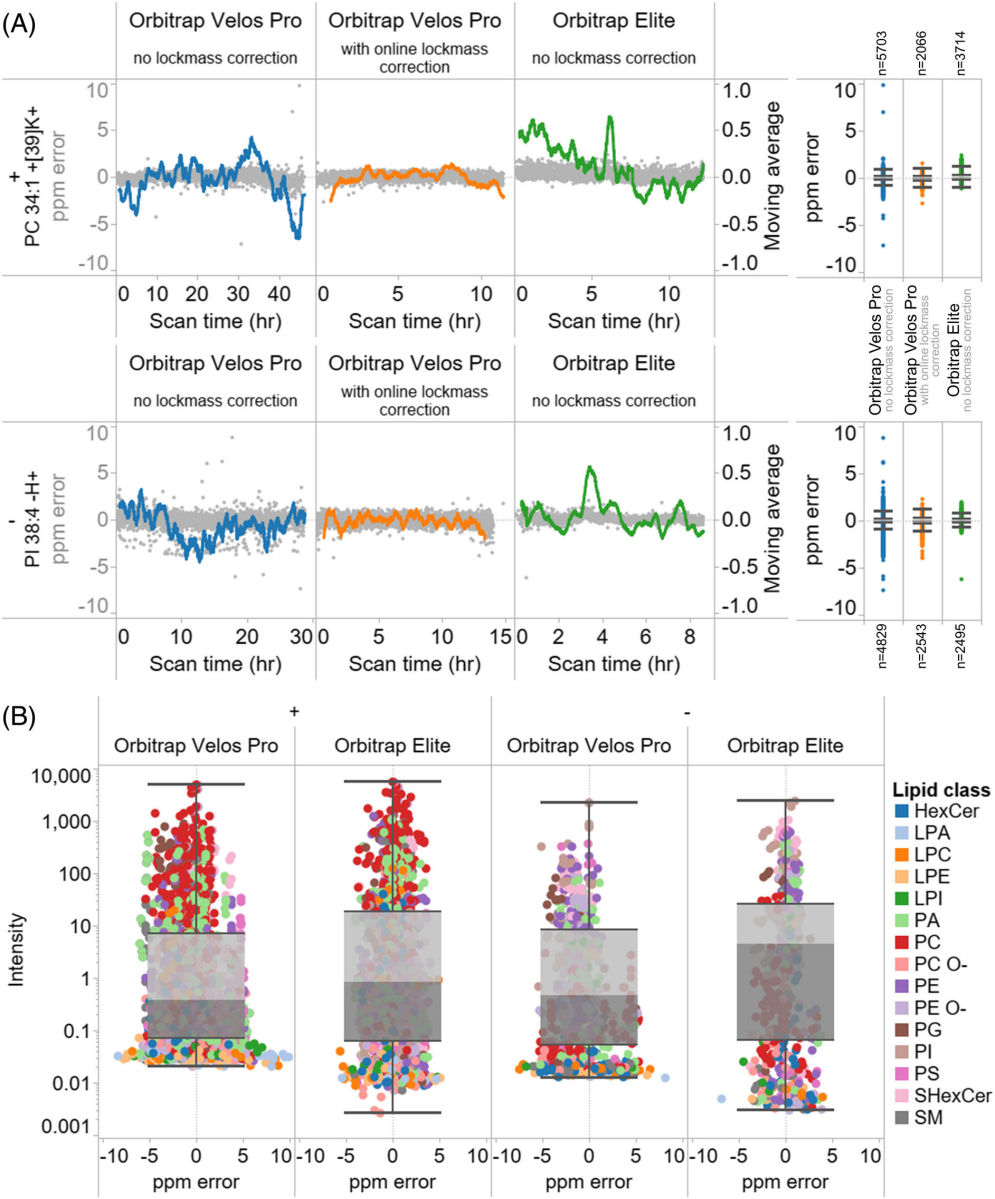
Assessment of FTMS calibration and dynamic detection range. (A) FTMS calibration as function of acquisition time with or without online lock‐mass correction for data recorded on the Orbitrap Velos Pro instrument. Data recorded on the Orbitrap Elite machine^14^ is shown for comparison. Measured ppm errors across all scans for potassiated PC 34:1 in positive ion mode and deprotonated PI 38:4 in negative ion mode are shown as individual dots. The moving average of ppm error, considering the last 100 scan events, is shown as a colored line. Note that the left y‐axis spans ±10 ppm and represents individual values (i.e., dots) whereas the right y‐axis spans ±1 ppm and represents the moving averages. (B) FTMS precursor ion intensity as function of ppm error for the 113 confidently identified lipid molecules reported by Ellis et al.^14^ Data values derive from five DDA‐imaging files recorded on the Orbitrap Velos Pro machine using online lock‐mass correction as well as three files recorded on the Orbitrap Elite machine.

For details related to hardware, we noted that the longer FTMS^1^ scan time of the Orbitrap Velos Pro would inevitably increase the scan time by 2.3‐fold per pixel and that this would propagate to a total of ~28 h when imaging greater tissue areas (Figure 1), as required for making tissue atlases. Based on this, we rationalized that the utility of the DDA‐imaging technology could be compromised by drifts in FTMS calibration over long acquisition times and that this could adversely impact lipid identification and image quality, both of which benefit from narrow ppm‐based tolerance windows. To examine this issue in further detail we monitored the accuracy of FTMS calibration by following the ppm‐error of selected lipid analytes across more than 28 h of DDA‐imaging on the Orbitrap Velos Pro machine (Figure 2A). This analysis showed that the ppm‐error, and hence the FTMS calibration, is relatively stable over long acquisition times in both positive and negative ion mode. Nevertheless, the analysis also revealed that the FTMS calibration drifts systematically on shorter timescales across a range of 1.1 ppm in positive and 0.8 ppm in negative ion mode (Figure 2A). Importantly, this performance is comparable with that of the Orbitrap Elite instrument, which exhibits almost identical calibration drifts, that is, 0.9 ppm and 0.8 ppm in positive and negative ion mode, respectively (Figure 2A).

To reduce these shorter term calibration drifts, we investigated the possibility of applying online FTMS lock mass calibration using the omnipresent dopant norharmane as a calibrant. This reduced the calibration drift to a narrower range of only 0.5 ppm in positive and 0.7 ppm in negative ion mode (Figure 2A). Notably, using online lock mass calibration also reduced the number of FTMS scans with ppm‐errors beyond ±1 ppm. In summary, in terms of FTMS mass accuracy and calibration stability, the DDA‐imaging method executed on the Orbitrap Velos Pro machine is comparable with that of the Orbitrap Elite.

We also evaluated the analytical sensitivity of the DDA‐method, since this impact both the overall lipidome coverage and the number of confidently identified lipid molecules that can be visualized. To this end, we benchmarked the dynamic range of FTMS^1^ analysis on the Orbitrap Velos Pro to that of the Orbitrap Elite by monitoring precursor ion intensities corresponding to the 113 confidently identified lipid molecules reported by Ellis et al.^14^ To our surprise, we found that DDA‐imaging on the Orbitrap Velos Pro is about 3‐fold less sensitive than on the Orbitrap Elite in both positive and negative ion mode (Figure 2B). This difference will inevitably result in lower lipidome coverage of DDA‐imaging when compared with the Orbitrap Elite.

Our systematic effort to transfer the DDA‐imaging technology also pinpointed other important technical parameters to be aware of. For example, optimizing the ion transmission settings with lipid standards instead of non‐lipid analytes present in commonly used calibration mixtures yielded a two‐fold increase in overall lipid signal intensity (data not shown). We also found that it was best to use lipid standards, and not non‐lipid calibration mixtures, for optimization of collision energy settings for DDA‐ITMS^2^ analysis (data not shown). Another beneficial feature was to use a DDA‐ITMS^2^ inclusion list to emulate sequential parallel reaction monitoring of all precursor *m/z* values across an *m/z* range 400–2000 and within an ion isolation window of ±0.5 Da.^20^ The benefit of this amendment is that that ITMS^2^ scans will always be acquired at the same precursor *m/z* value (e.g., *m/z* 760.58) and prompt only one unique “scan header” in proprietary. RAW mass spectral data files instead of multiple practically identical “scan headers” (e.g., *m/z* 760.57, 760.58, and 760.59). Our approach thereby makes it is easier to manually inspect ITMS^2^ spectra with the QualBrowser software and, more importantly, identical ITMS^2^ scan events will be averaged together improving the signal‐to‐noise of detected fragment ions.

### 3.2 Benchmarking the DDA‐imaging technology

**FIGURE 3:**
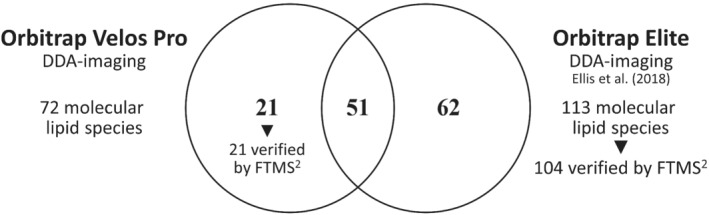
Benchmarking lipid identifications obtained by DDA‐imaging on the Orbitrap Velos Pro and the Orbitrap Elite. Confident lipid identifications were obtained from five positive and five negative DDA‐imaging experiments of mouse brain carried out using the Orbitrap Velos Pro and three positive and three negative DDA‐imaging experiments of rat brain using the Orbitrap Elite. Detected lipid molecules were identified using the ALEX^123^ software. Venn diagram displays the lipidome overlap between the two DDA‐imaging platforms. The 21 unique lipid identifications obtained using the Orbitrap Velos Pro were validated by high‐resolution FTMS^2^ analysis using an Orbitrap Fusion^20^ (see Figure S1).

Having implemented the DDA‐imaging technology on the Orbitrap Velos Pro, we next evaluated its performance in terms of lipidome coverage. To this end, we analyzed five mouse brain sections in both positive and negative ion mode and used ALEX^123^ software to automatically identify individual lipid molecules. This analysis identified 72 lipid molecules at the molecular species‐level (i.e., with annotation of the number of C atoms, double bonds and hydroxyl‐groups in individual hydrocarbon chains) (Figure 3). This coverage is less than that obtained using the Orbitrap Elite, which covered 113 molecular lipid species. The lower lipidome coverage corroborates the notion that the Orbitrap Velos Pro‐based platform is indeed less sensitive. Nevertheless, DDA‐imaging on the Orbitrap Velos Pro was able to recapitulate 51 lipid identification previously identified using the Orbitrap Elite. Furthermore, the Orbitrap Velos Pro‐based platform shortlisted 21 unique lipid identifications, which could be verified by manual inspection of high‐resolution FTMS^2^ spectra of mouse brain tissue recorded using an Orbitrap Fusion (Figure S1).^20^ Notably, majority of these lipid annotations are borderline identifications in the Orbitrap Elite dataset (Figure S1), rejected originally due to strict constraints.

These results, together with the assessments of instrument hardware, sensitivity and FTMS^1^ calibration stability, demonstrate that the performance of DDA‐imaging on the Orbitrap Velos Pro is inferior to that on the Orbitrap Elite instrument. Nevertheless, it appears to be relatively easy to transfer the DDA‐imaging technology from one system to another.

### 3.3 Reproducible DDA‐imaging of brain lipids

**FIGURE 4:**
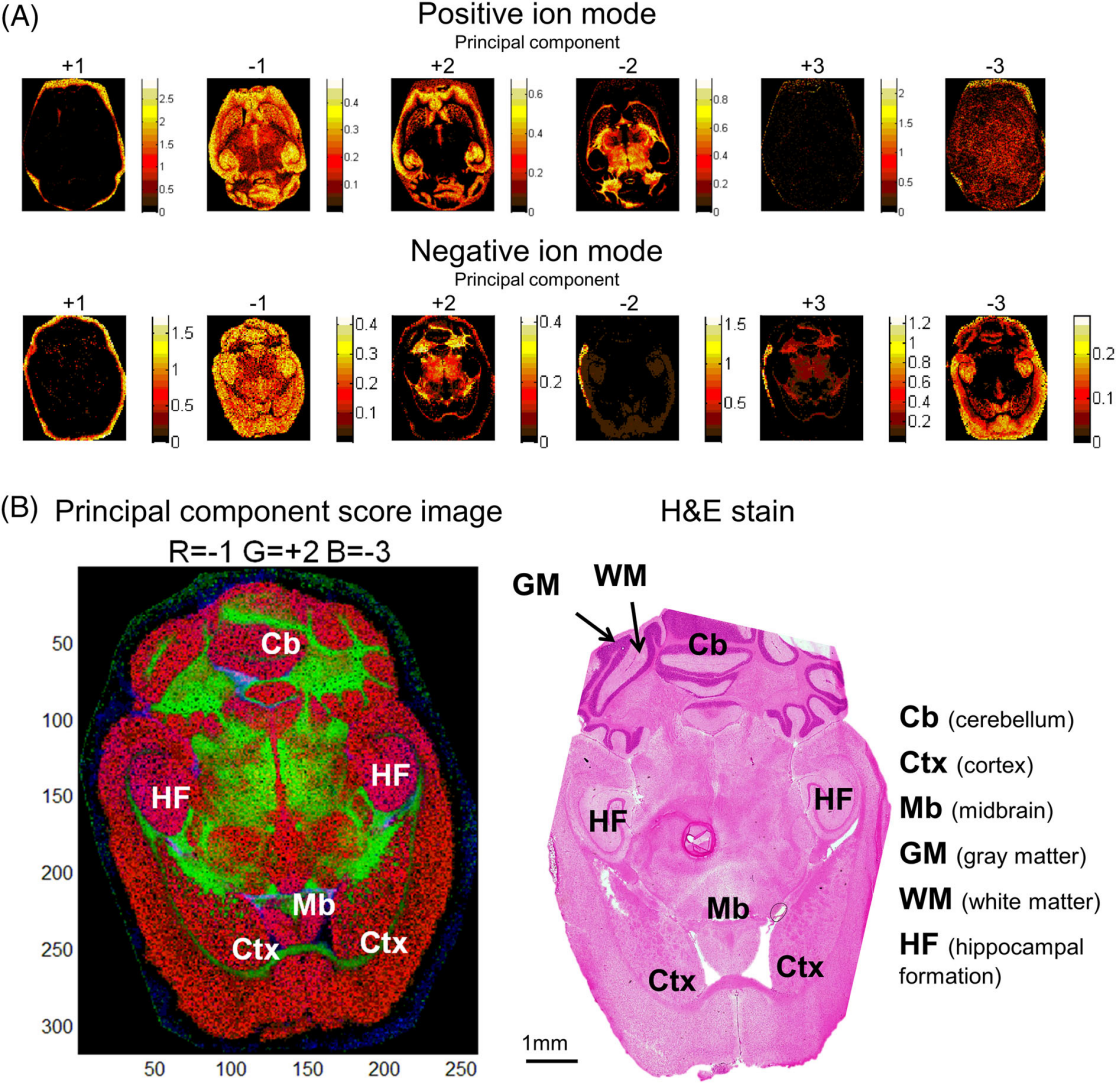
Identification of lipid molecules with significant differences in spatial distribution. (A) Image segmentation by principal component analysis of representative DDA‐image acquired in positive and negative ion mode. (B) Correlation of optical image of H&E stained tissue section and a principal component score‐based image. The DDA‐imaging data were recorded in positive ion mode. Red color represents principal component −1, green color represents principle component +2 and blue color represents principle component −3.

Next, we assessed the performance of the DDA‐imaging technology in terms of its ability to reproducibly generate images of lipid molecules across multiple tissue sections as required for making tissue atlases from consecutive sections. To this end, we first identified lipid molecules displaying the most pronounced differences in distribution across the mouse brain. Our aim was to utilize such molecular ions as markers to assess image reproducibility across consecutive tissue sections. To identify such lipid markers, we performed principal component analysis of representative FTMS^1^ ion images in positive and negative ion mode (Figures 4A and S2A). This analysis shortlisted 22 distinct lipid molecules that display major differences in topology (Figure S2B). These biomarkers are primarily glycerophospholipids, including phosphatidylcholine (PC) and phosphatidylethanolamine (PE) species, and sphingolipids, including sphingomyelin (SM), hexosylceramide (HexCer), and sulfatide (SHexCer) species as well as a putative ganglioside. Importantly, we found that images obtained by DDA‐imaging in both polarities showed a close correlation to the underlying mouse brain anatomy assessed by conventional H&E staining and optical microscopy (Figure 4B).

### 3.4 Reproducible DDA‐imaging of glycerophospholipids

**FIGURE 5:**
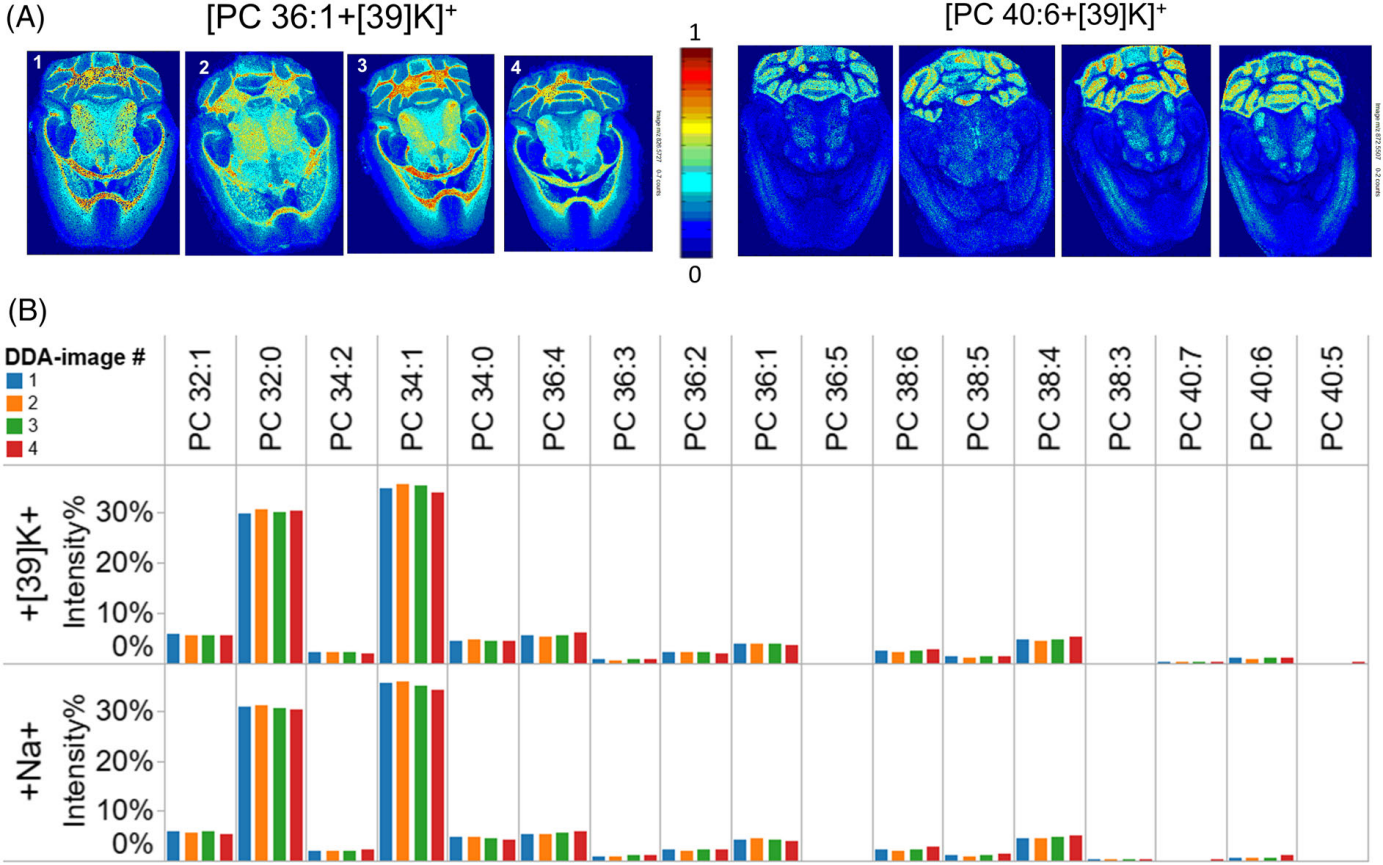
Reproducibility of DDA‐imaging of PC species across multiple brain sections. (A) Ion images of potassiated PC 36:1(18:0‐18:1) and PC 40:6(18:0‐22:6) across four distinct brain sections. (B) FTMS^1^ intensity profile of PC species with different adduct ions across four brain sections.

To examine more closely the reproducibility of the lipid imaging, we first inspected the topology of the shortlisted glycerophospholipid markers PC 36:1 and PC 40:6 across four horizontal brain sections. Notably, ALEX^123^ automatically identified these as the molecular lipid species PC 18:0‐18:1 and PC 18:0‐22:6, based on the detection of FA chain‐specific fragments in the parallel ITMS^2^ analysis. Inspecting ion images for PC 18:0‐18:1(36:1) and PC 18:0‐22:6(40:6) showed a good reproducibility across the four individual brain sections (Figure 5A), where the former is most abundant in white matter of the cerebellum, the midbrain and caudate putamen, and the latter is most abundant in gray matter of the cerebellum (Figure 5A). As a complementary proxy for assessing the image quality and reproducibility we also examined the FTMS^1^ intensity distribution across all identified PC molecules (Figure 5B). This demonstrated that the DDA‐imaging routine across multiple tissue sections produces highly reproducible profile of glycerophospholipid ionization, which underpin the utility of DDA‐method as a robust tool for cross‐sectional lipid imaging.

### 3.5 Reproducible DDA‐imaging of sphingolipids

**FIGURE 6:**
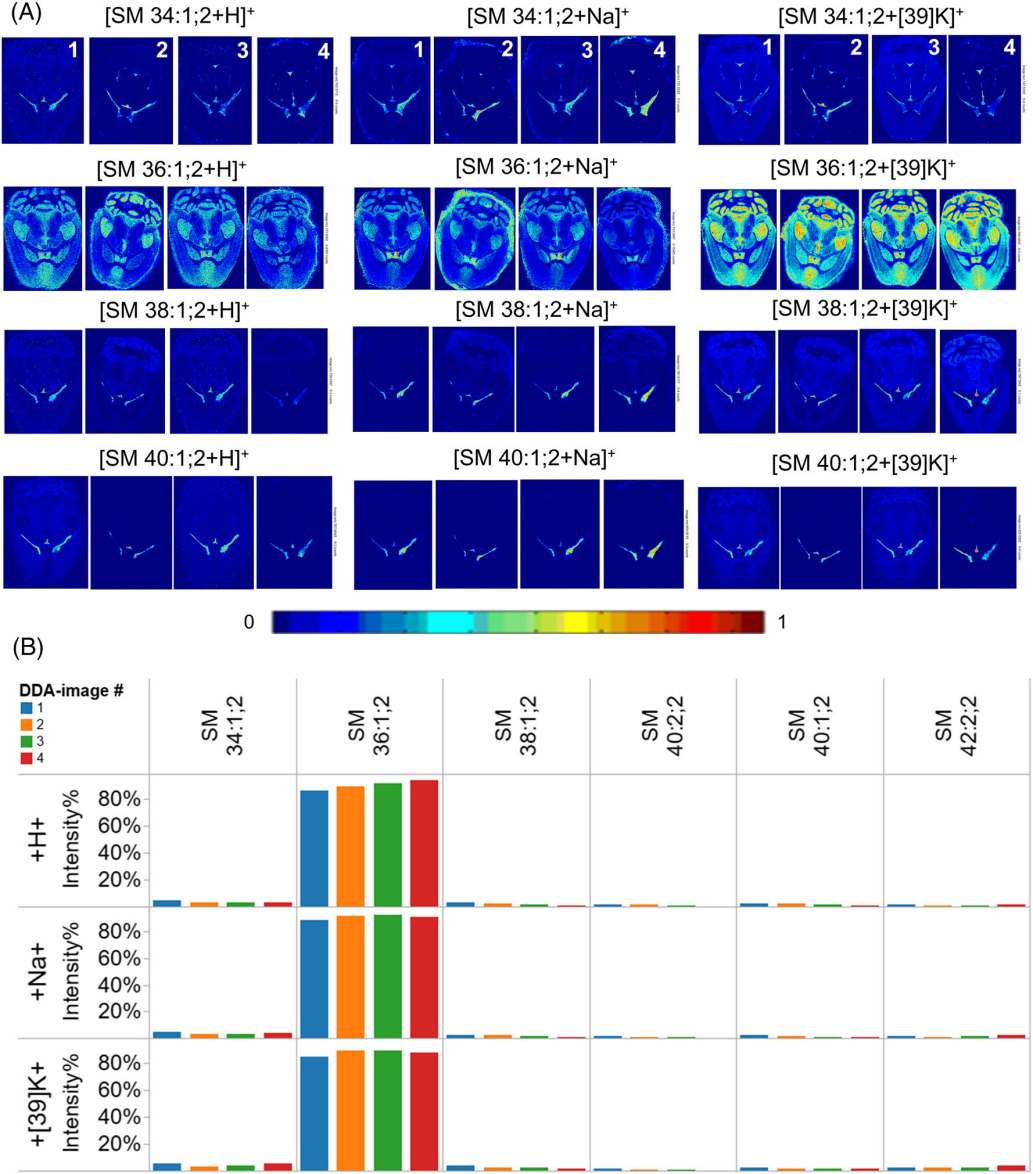
Reproducibility of DDA‐imaging of SM species across multiple brain sections. (A) Ion images of protonated, sodiated and potassiated SM species across four distinct brain sections. (B) FTMS^1^ intensity profile of SM species with different adduct ions across the four brain sections.

We also examined the reproducibility of DDA‐imaging of sphingolipids, given that these molecules generally yield less intense intensities as compared to more abundant glycerophospholipids. Among the sphingolipids with major differences in localization we identified four distinct SM molecules: SM 34:1;2, SM 36:1;2, SM 38:1;2, and SM 42:1;2. Notably, the parallel ITMS^2^ analysis of these lipids only yielded detection of the confirmatory lipid class‐specific phosphocholine fragment ion with *m/z* 184.1 and no long‐chain base‐specific fragments that allows inferring the structure of the ceramide‐backbone (e.g., *m/z* 264.3).^19^ Nevertheless, the topology of these SM species again demonstrated a good reproducibility across the four brain sections and for several adduct ions (Figure 6A). Notably, we found that the most abundant SM 36:1;2 localizes to areas of gray matter throughout the brain whereas the three other SM species are more abundant in the choroid plexus. When assessing the FTMS^1^ intensity distribution across all identified SM molecules we again observed a highly reproducible profile of ionization for protonated, sodiated as well as potassiated precursor ions (Figure 6B).

### 3.6 DDA‐imaging of gangliosides

**FIGURE 7:**
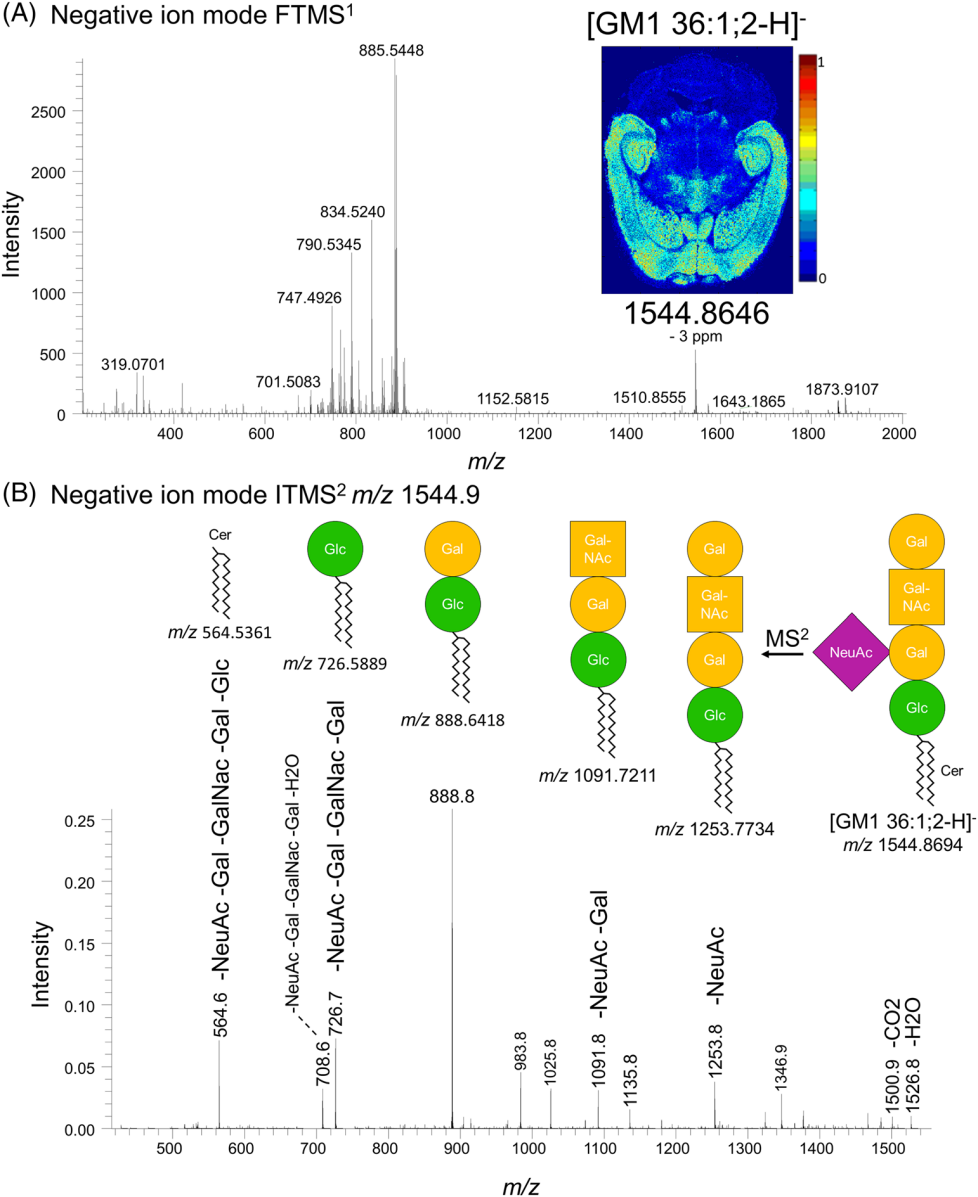
DDA‐imaging and structural characterization of ganglioside GM1 36:1;2. (A) Negative ion mode FTMS spectrum showing detection of deprotonated GM1 36:1;2 at *m/z* 1544.8646. The insert shows the ion image for *m/z* 1544.8646. (B) Negative ion mode ITMS^2^ spectrum of *m/z* 1544.9 with annotation of GM1 36:1;2‐derived fragment ions.

Our principal component analysis‐based search for topology markers also yielded a prominent molecular ion with *m/z* 1544.8615 (Figures 7A and S2B). By manual inspection of the DDA‐imaging data we elucidated that this ion is a deprotonated, singly charged ganglioside with the species‐level annotation GM1 36:1;2 (detected with mass accuracy error of −3 ppm). This identification was further supported by the parallel ITMS^2^ analysis of *m/z* 1544.9 showing multiple characteristic neutral loss fragments corresponding to the oligosaccharide‐based head group structure (Figure 7B). Unfortunately, no long‐chain base‐ or FA‐specific fragment ions were detected to provide insights into the molecular structure of the ceramide‐backbone. Nevertheless, it is reasonable to predict that the molecular species is primarily GM1 18:1;2/18:0, since numerous reports have previously shown that majority of brain sphingolipids have a backbone composed of a C18 sphingosine with an amide‐linked C18 acyl chain.^21^ Finally, the ion image shows that GM1 36:1;2 is most abundant in the cerebral cortex, the hippocampus and the midbrain region of the mouse brain (Figure 7A).

## 4 CONCLUSIONS

Here, we outlined the successful transfer and implementation of DDA‐imaging technology on an Orbitrap Velos Pro machine and benchmarked its performance for lipidome imaging across multiple consecutive tissue sections. Overall, our report provides a guideline and insights into various technical issues that warrant special attention when implementing the technology on another type of mass spectrometer. More specifically, we found that DDA‐imaging on an Orbitrap Velos Pro is comparable with that of the Orbitrap Elite in terms of ease‐of‐operation, lipid image reproducibility and ability to shortlist differentially localized lipid molecules such as GM1, which was not identified in the original study by Ellis et al.^14^ Moreover, we show that DDA‐imaging on the Orbitrap Velos Pro was able to identify half of the confidently annotated molecular lipid species reported previously as well as another 21 lipid molecules that could be verified by high‐resolution shotgun lipidomic analysis of mouse brain extracts and that were previously considered borderline identifications in the Orbitrap Elite dataset. Our work also indicated several drawbacks of using an Orbitrap Velos Pro, which includes an inherently lower resolution, slower scan rate, lower sensitivity and dynamic FTMS detection range, which concur with an overall lower lipidome coverage. We note that other technical parameters could in principle have affected the comparison of the two platforms. These include the animal species used for making tissue sections (rat cerebellum was used for benchmarking the Orbitrap Elite‐based platform), the thickness of tissue sections (herein we used 15 μm thick sections whereas the rat cerebellar tissue was 10 μm thick) and whether tissues are obtained following whole‐body perfusion to remove blood contamination (as was done herein). To the best of our knowledge, these parameters are, however, of minor relevance for showcasing the overall performance metrics of DDA‐imaging on the Orbitrap Velos Pro machine. In summary, based on our systematic performance assessment we conclude that Orbitrap Velos Pro serves as a robust tool for routine lipid imaging, covering a limited number of tissue sections, as well for more challenging and time‐consuming endeavors such as making tissue atlases, which can entail analysis of hundreds of consecutive tissue sections.

## CONFLICT OF INTEREST

The authors declare no conflict of interest.

## AUTHOR CONTRIBUTIONS

CSE conceived the study. CSE, SMK, and RRS performed the experiments. SMK prepared the tissue samples. CSE, SMK, and RRS analyzed the data. CSE wrote the manuscript. CSE and MH supervised the project.

## Supporting information


**Figure S1.**Validation of 21 unique lipid molecules by comparison of MS^2^ data recorded by DDA‐imaging on the Orbitrap Velos Pro and the Orbitrap Elite as well as by shotgun MS^ALL^ analysis of 16 mouse brain lipid extracts on an Orbitrap Fusion.

**Figure S2.** Identification of lipid molecules with major differences in spatial distribution. (A) Loading spectra for the first three principal components for the positive and negative DDA‐images in Figure 4A. (B) List of lipid molecules with most pronounced differences in topology.

## Data Availability

The data that support the findings of this study are available from the corresponding author upon reasonable request.
