# Supplementary material for: DDA‐imaging with structural identification of lipid molecules on an Orbitrap Velos Pro mass spectrometer
Source: J Mass Spectrom. 2022 Sep 2;57(9):e4882. doi: 10.1002/jms.4882 (PMC9541402; doi:10.1002/jms.4882)
Supplement: Supplementary file 1 — Figure S1.Validation of 21 unique lipid molecules by comparison of MS2 data recorded by DDA‐imaging on the Orbitrap Velos Pro and the Orbitrap Elite as well as by shotgun MSALL analysis of 16 mouse brain lipid extracts on an Orbitrap Fusion. [file JMS-57-e4882-s001.pdf]

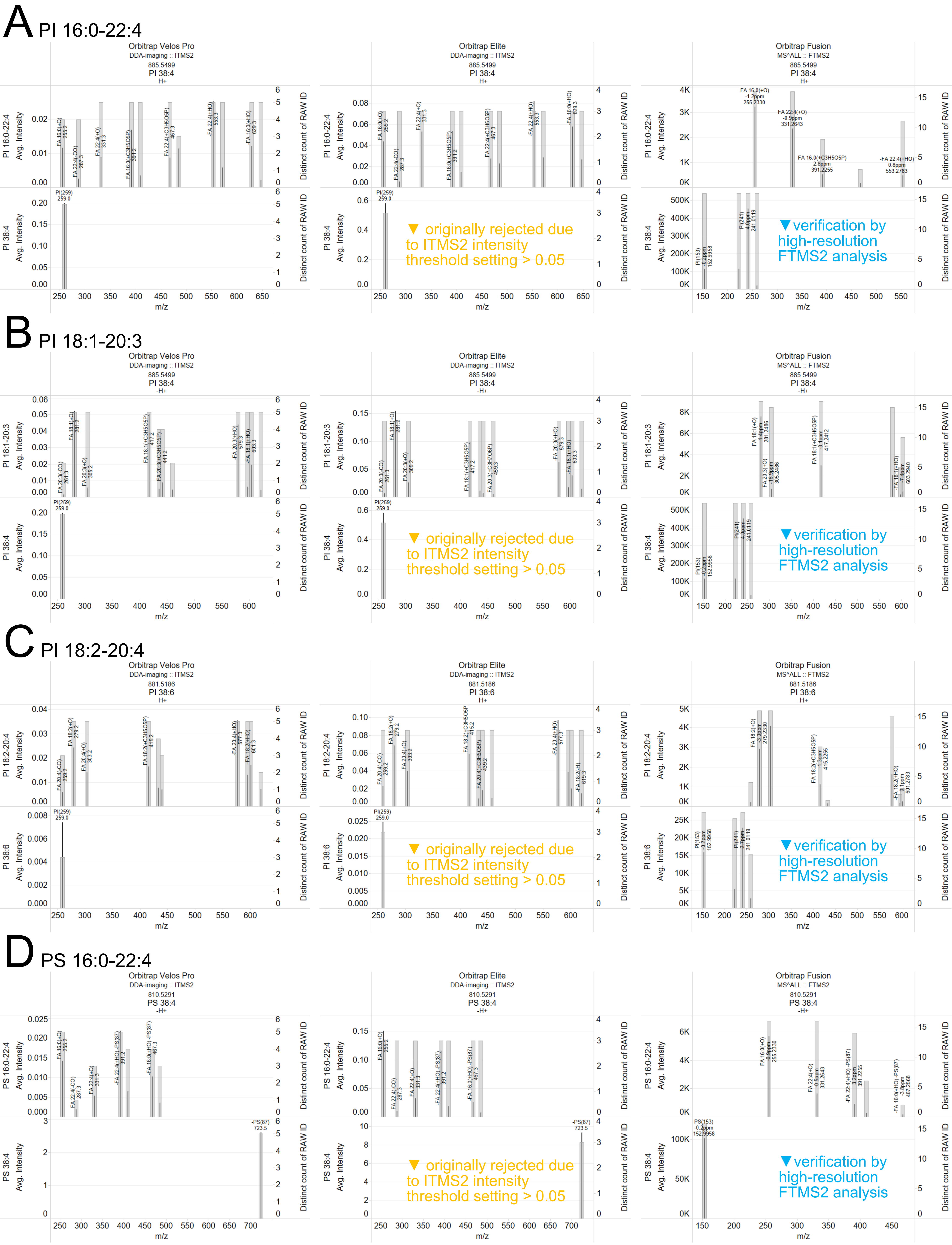

A PI 16:0-22:4
B PI 18:1-20:3
C PI 18:2-20:4
D PS 16:0-22:4
Orbitrap Velos Pro
DDA-imaging :: ITMS2
Orbitrap Elite
DDA-imaging :: ITMS2
Orbitrap Fusion
MS^ALL :: FTMS2
885.5499
PI 38:4
881.5186
PI 38:6
810.5291
PS 38:4
-H+
Avg. Intensity
Distinct count of RAW ID
m/z
▼ originally rejected due to ITMS2 intensity threshold setting > 0.05
▼ verification by high-resolution FTMS2 analysis

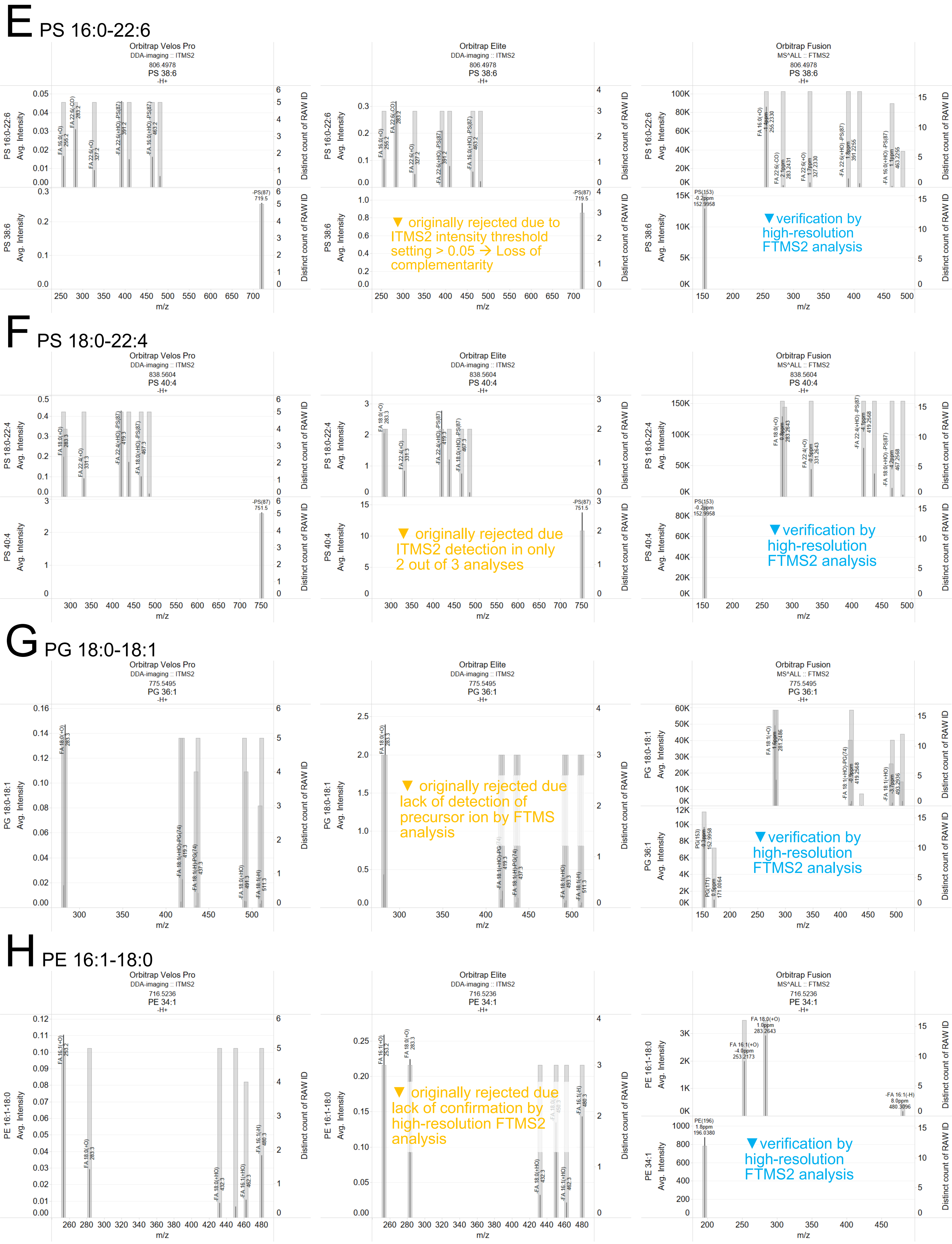

E PS 16:0-22:6
Orbitrap Velos Pro
DDA-imaging :: ITMS2
806.4978
PS 38:6
Orbitrap Elite
DDA-imaging :: FTMS2
Orbitrap Fusion
MS^ALL :: FTMS2
▼ originally rejected due to ITMS2 intensity threshold setting > 0.05 → Loss of complementarity
▼ verification by high-resolution FTMS2 analysis
F PS 18:0-22:4
Orbitrap Velos Pro
DDA-imaging :: ITMS2
838.5604
PS 40:4
Orbitrap Elite
DDA-imaging :: ITMS2
Orbitrap Fusion
MS^ALL :: FTMS2
▼ originally rejected due ITMS2 detection in only 2 out of 3 analyses
▼ verification by high-resolution FTMS2 analysis
G PG 18:0-18:1
Orbitrap Velos Pro
DDA-imaging :: ITMS2
775.5495
PG 36:1
Orbitrap Elite
DDA-imaging :: ITMS2
Orbitrap Fusion
MS^ALL :: FTMS2
▼ originally rejected due lack of detection of precursor ion by FTMS analysis
▼ verification by high-resolution FTMS2 analysis
H PE 16:1-18:0
Orbitrap Velos Pro
DDA-imaging :: ITMS2
716.5236
PE 34:1
Orbitrap Elite
DDA-imaging :: ITMS2
Orbitrap Fusion
MS^ALL :: FTMS2
▼ originally rejected due lack of confirmation by high-resolution FTMS2 analysis
▼ verification by high-resolution FTMS2 analysis
Avg. Intensity
Distinct count of RAW ID
m/z

I PE 18:1-20:3

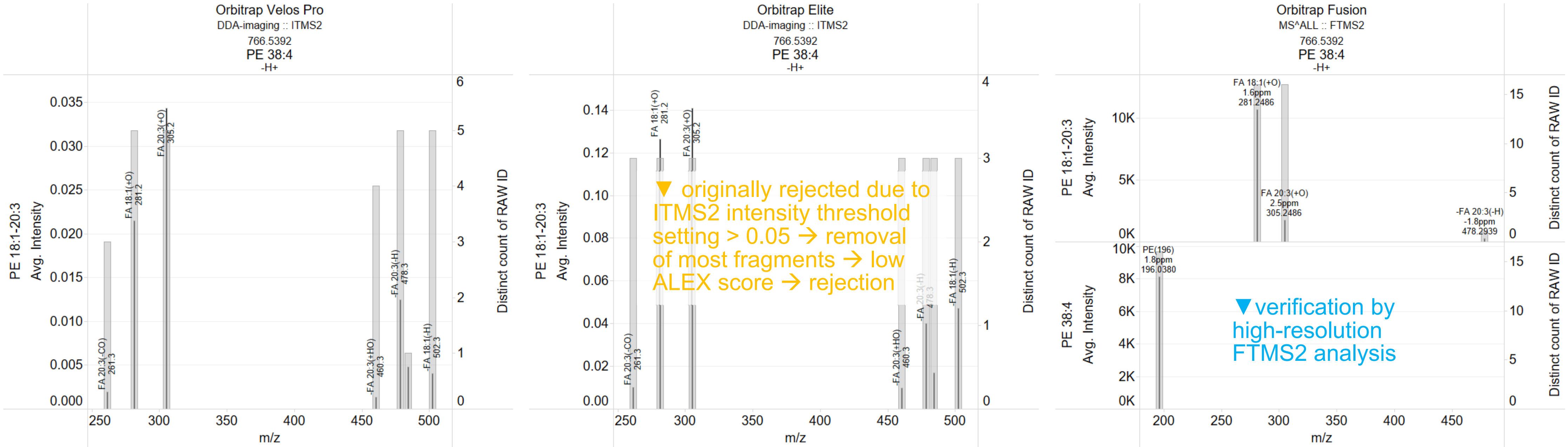


J PE 16:0-22:5

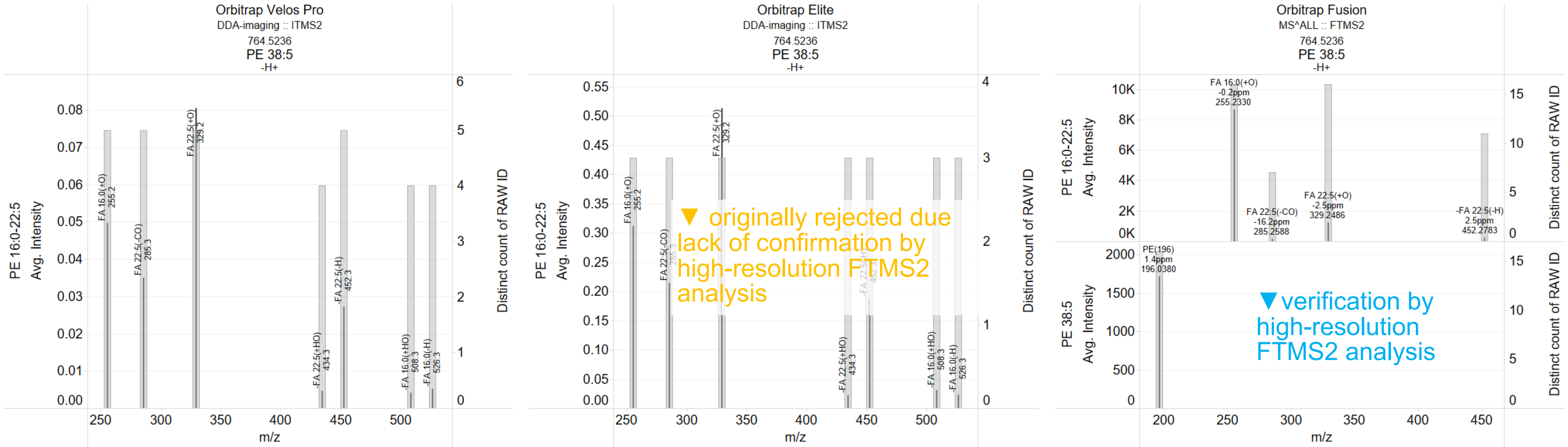


K PE O-20:0/18:1

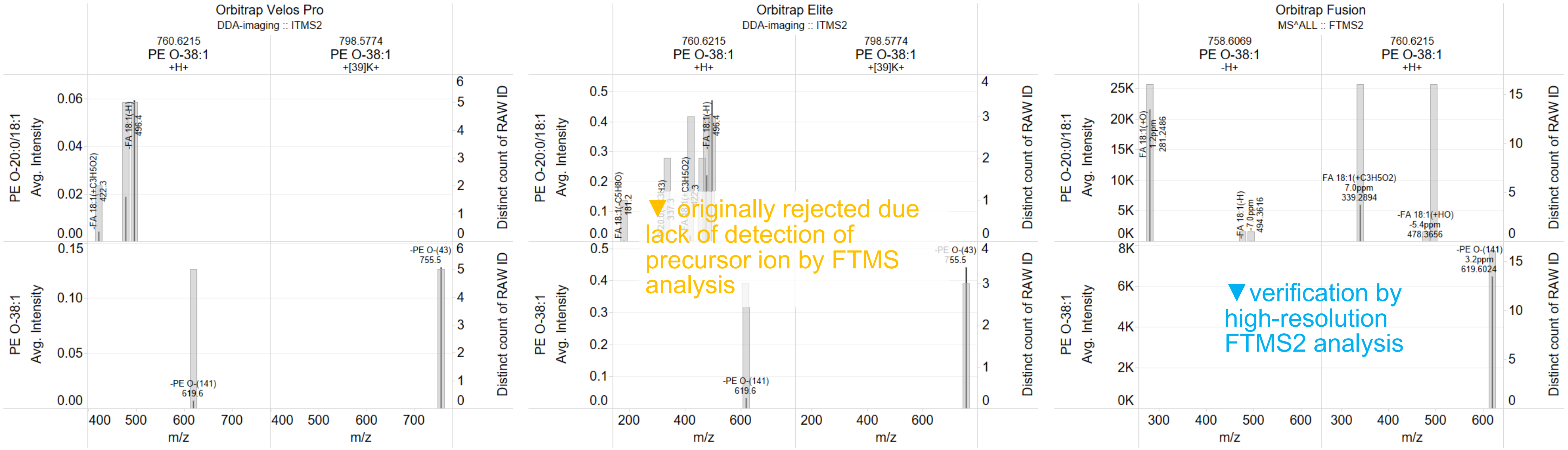


L PE O-22:1/16:0

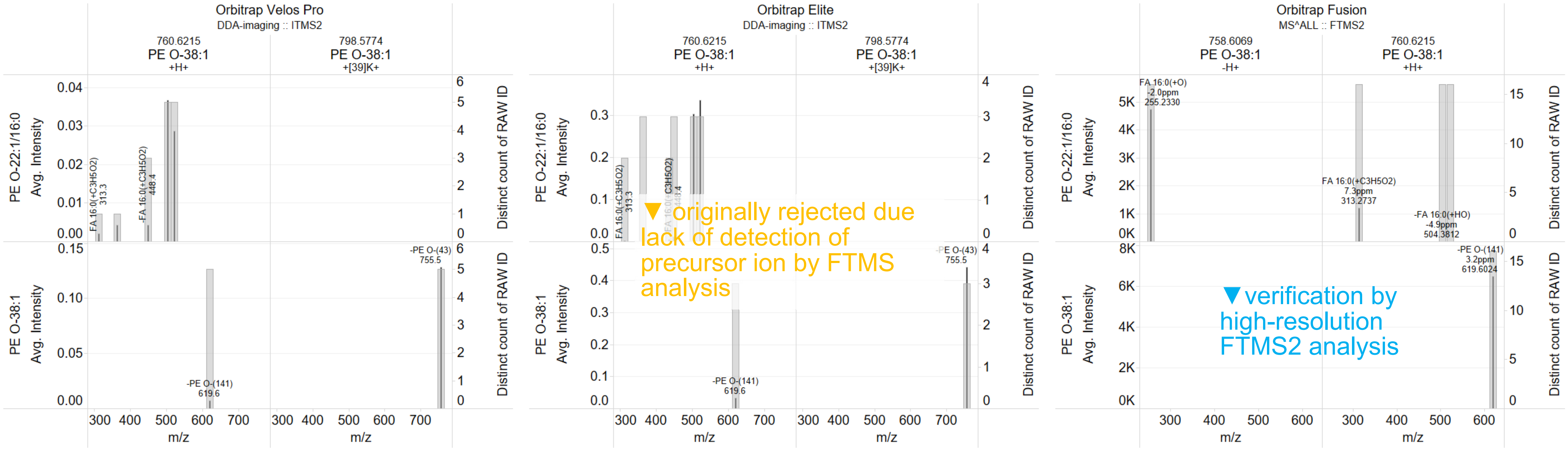

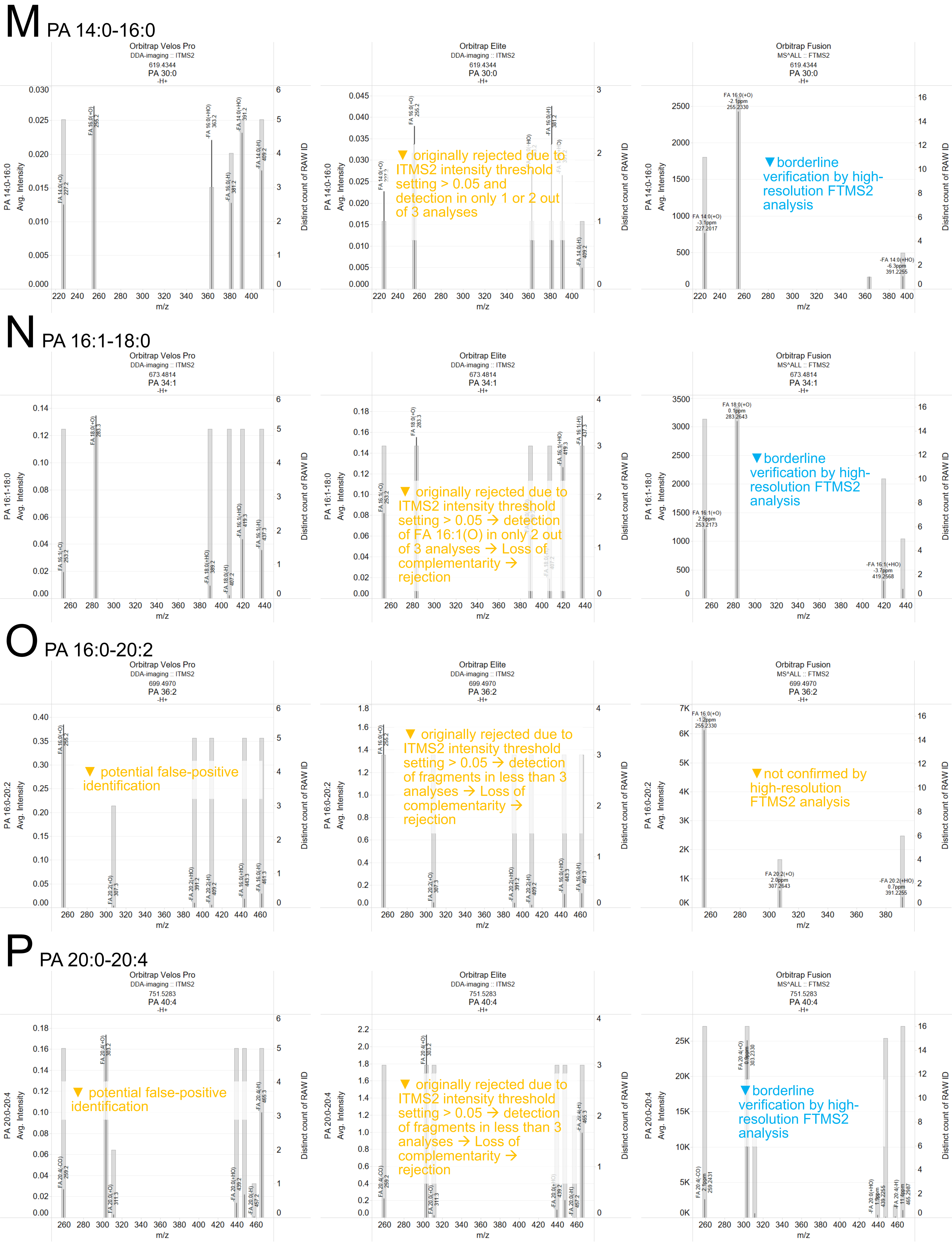
M PA 14:0-16:0
Orbitrap Velos Pro
DDA-imaging :: ITMS2
619.4344
PA 30:0
-H+
Orbitrap Elite
DDA-imaging :: ITMS2
619.4344
PA 30:0
-H+
▼ originally rejected due to ITMS2 intensity threshold setting > 0.05 and detection in only 1 or 2 out of 3 analyses
Orbitrap Fusion
MS^ALL :: FTMS2
619.4344
PA 30:0
-H+
▼borderline verification by high-resolution FTMS2 analysis
FA 16:0(+O) -2.1ppm 255.2330
FA 14:0(+O) -3.1ppm 227.2017
-FA 14:0(+HO) -6.3ppm 391.2255
N PA 16:1-18:0
Orbitrap Velos Pro
DDA-imaging :: ITMS2
673.4814
PA 34:1
-H+
Orbitrap Elite
DDA-imaging :: ITMS2
673.4814
PA 34:1
-H+
▼ originally rejected due to ITMS2 intensity threshold setting > 0.05 → detection of FA 16:1(O) in only 2 out of 3 analyses → Loss of complementarity → rejection
Orbitrap Fusion
MS^ALL :: FTMS2
673.4814
PA 34:1
-H+
▼borderline verification by high-resolution FTMS2 analysis
FA 18:0(+O) 0.1ppm 283.2643
FA 16:1(+O) 2.5ppm 253.2173
-FA 16:1(+HO) -3.7ppm 419.2568
O PA 16:0-20:2
Orbitrap Velos Pro
DDA-imaging :: ITMS2
699.4970
PA 36:2
-H+
▼ potential false-positive identification
Orbitrap Elite
DDA-imaging :: ITMS2
699.4970
PA 36:2
-H+
▼ originally rejected due to ITMS2 intensity threshold setting > 0.05 → detection of fragments in less than 3 analyses → Loss of complementarity → rejection
Orbitrap Fusion
MS^ALL :: FTMS2
699.4970
PA 36:2
-H+
▼not confirmed by high-resolution FTMS2 analysis
FA 16:0(+O) -1.2ppm 255.2330
FA 20:2(+O) 2.0ppm 307.2643
-FA 20:2(+HO) 0.7ppm 391.2255
P PA 20:0-20:4
Orbitrap Velos Pro
DDA-imaging :: ITMS2
751.5283
PA 40:4
-H+
▼ potential false-positive identification
Orbitrap Elite
DDA-imaging :: ITMS2
751.5283
PA 40:4
-H+
▼ originally rejected due to ITMS2 intensity threshold setting > 0.05 → detection of fragments in less than 3 analyses → Loss of complementarity → rejection
Orbitrap Fusion
MS^ALL :: FTMS2
751.5283
PA 40:4
-H+
▼borderline verification by high-resolution FTMS2 analysis
Avg. Intensity
Distinct count of RAW ID
m/z

# Q PA 20:4-22:6

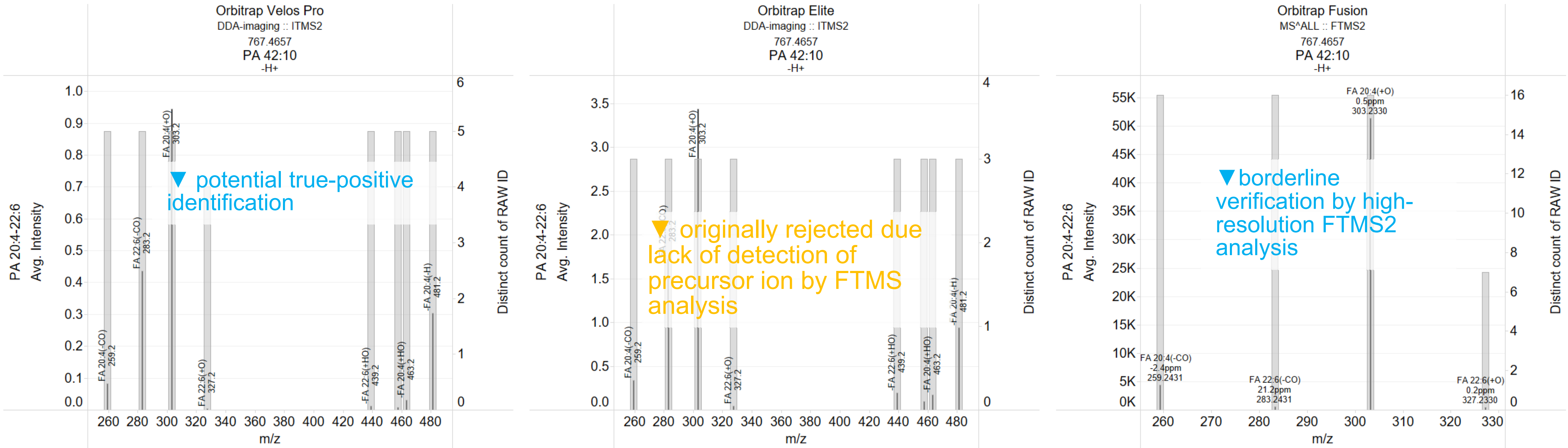


# R PA 22:5-22:6

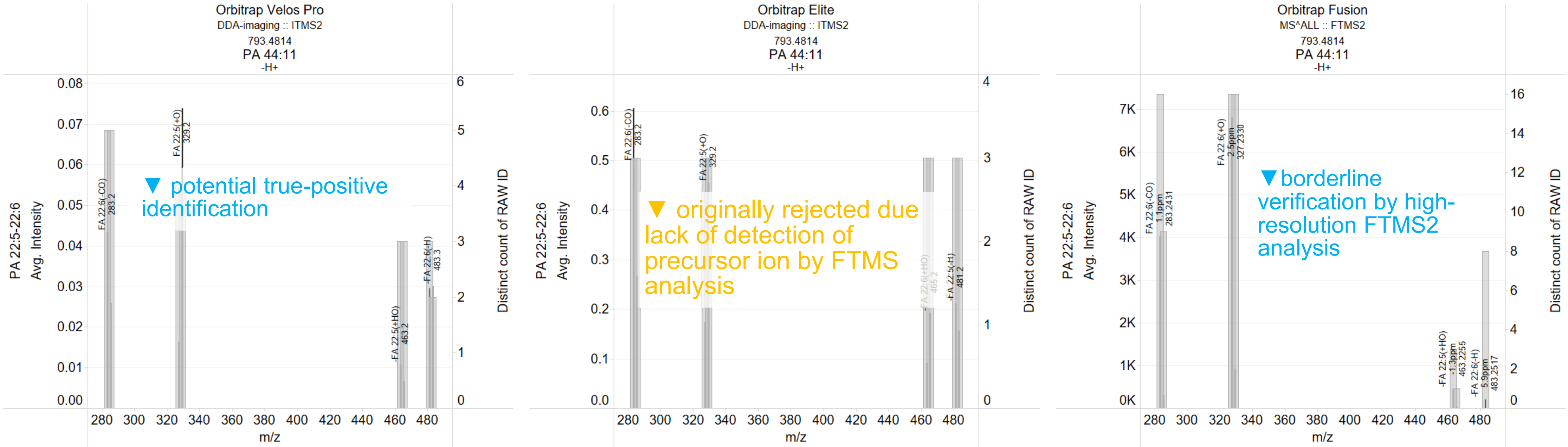


# S LPA 18:0

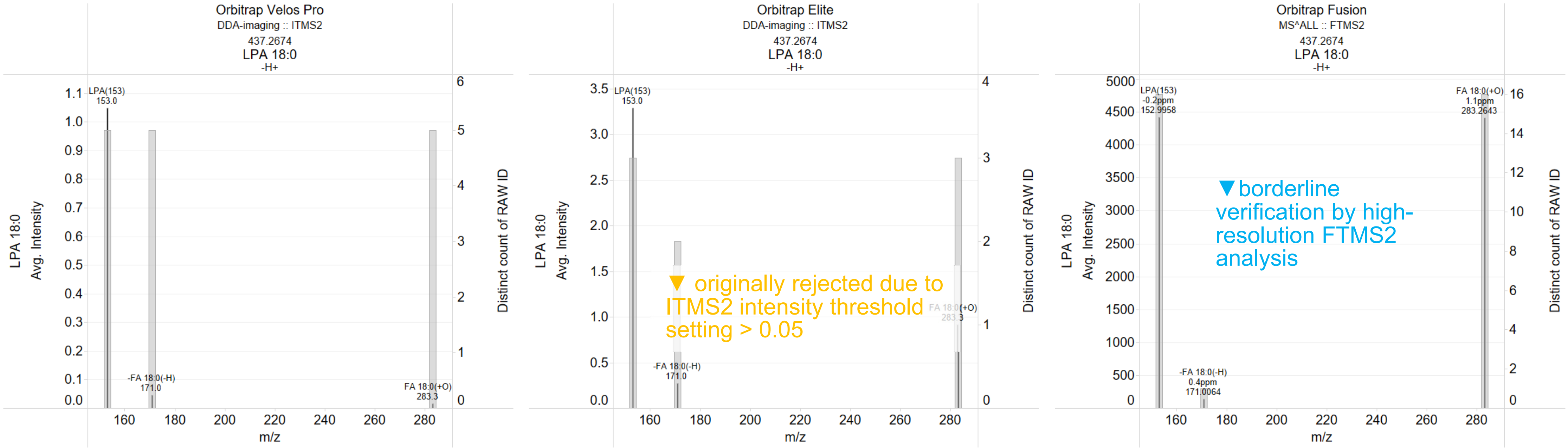


# T LPE O-16:0

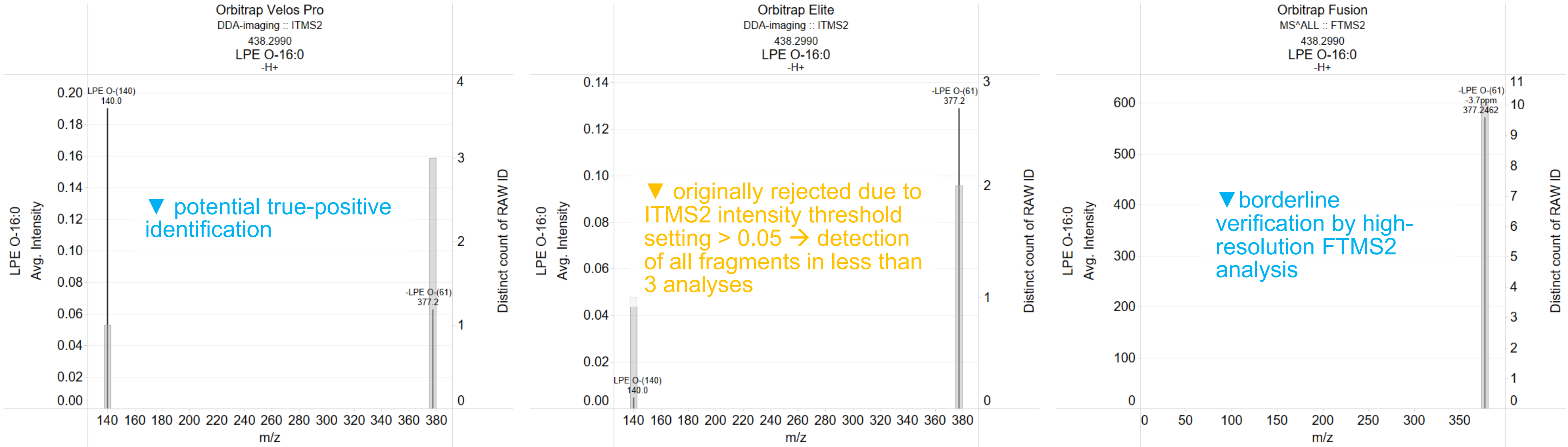

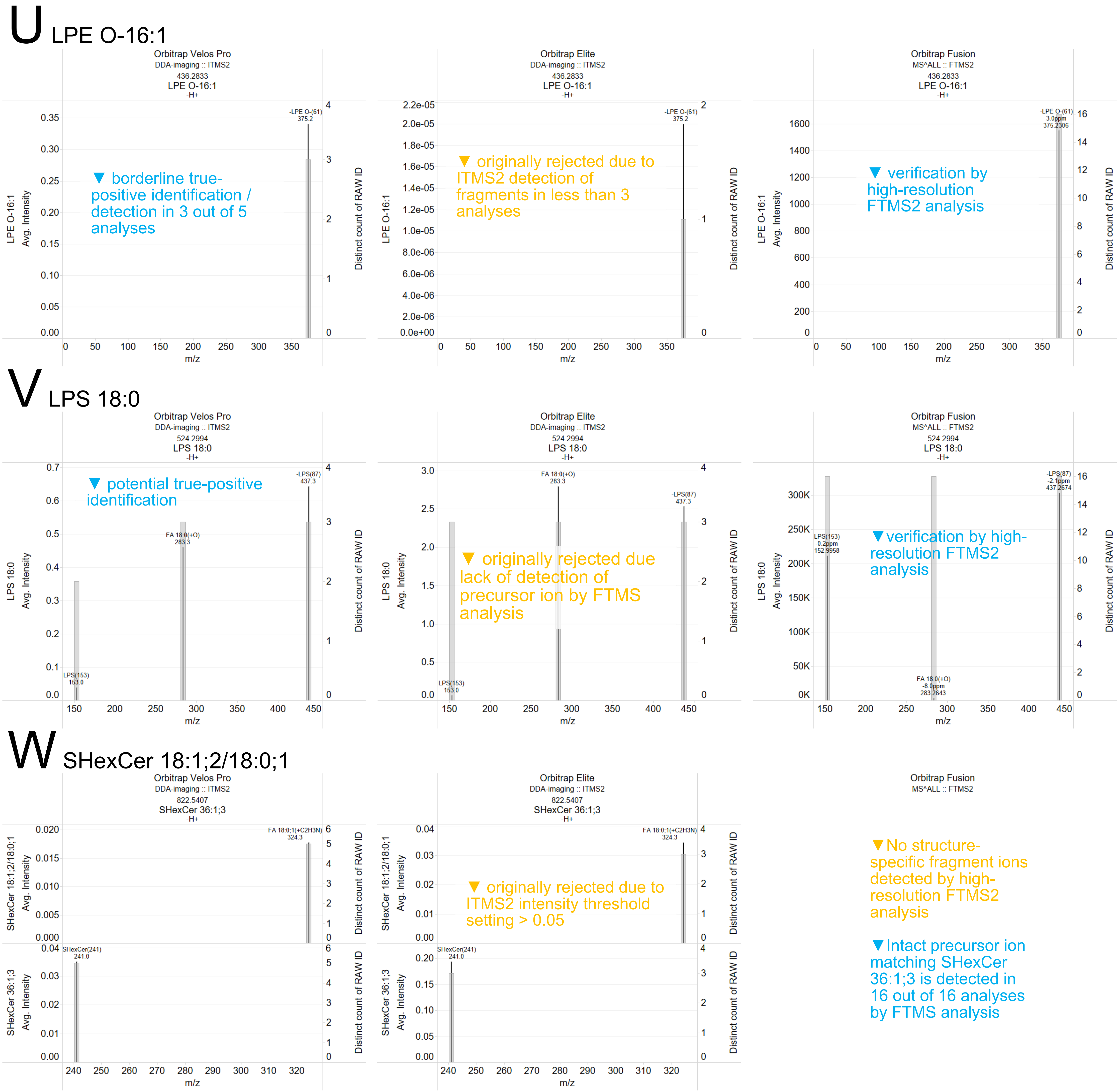

U LPE O-16:1
Orbitrap Velos Pro
DDA-imaging :: ITMS2
436.2833
LPE O-16:1
-H+
▼ borderline true-positive identification / detection in 3 out of 5 analyses
Orbitrap Elite
DDA-imaging :: ITMS2
▼ originally rejected due to ITMS2 detection of fragments in less than 3 analyses
Orbitrap Fusion
MS^ALL :: FTMS2
▼ verification by high-resolution FTMS2 analysis
-LPE O-(61) 375.2
-LPE O-(61) 3.0ppm 375.2306
LPE O-16:1 Avg. Intensity
Distinct count of RAW ID
m/z
V LPS 18:0
Orbitrap Velos Pro
DDA-imaging :: ITMS2
524.2994
LPS 18:0
-H+
▼ potential true-positive identification
Orbitrap Elite
DDA-imaging :: ITMS2
▼ originally rejected due lack of detection of precursor ion by FTMS analysis
Orbitrap Fusion
MS^ALL :: FTMS2
▼ verification by high-resolution FTMS2 analysis
LPS(153) 153.0
FA 18:0(+O) 283.3
-LPS(87) 437.3
LPS(153) -0.2ppm 152.9958
FA 18:0(+O) -8.0ppm 283.2643
-LPS(87) -2.1ppm 437.2674
LPS 18:0 Avg. Intensity
Distinct count of RAW ID
m/z
W SHexCer 18:1;2/18:0;1
Orbitrap Velos Pro
DDA-imaging :: ITMS2
822.5407
SHexCer 36:1;3
-H+
Orbitrap Elite
DDA-imaging :: ITMS2
▼ originally rejected due to ITMS2 intensity threshold setting > 0.05
Orbitrap Fusion
MS^ALL :: FTMS2
▼ No structure-specific fragment ions detected by high-resolution FTMS2 analysis
▼ Intact precursor ion matching SHexCer 36:1;3 is detected in 16 out of 16 analyses by FTMS analysis
FA 18:0;1(+C2H3N) 324.3
SHexCer(241) 241.0
SHexCer 18:1;2/18:0;1 Avg. Intensity
SHexCer 36:1;3 Avg. Intensity
Distinct count of RAW ID
m/z
