# Supplementary material for: DDA‐imaging with structural identification of lipid molecules on an Orbitrap Velos Pro mass spectrometer
Source: J Mass Spectrom. 2022 Sep 2;57(9):e4882. doi: 10.1002/jms.4882 (PMC9541402; doi:10.1002/jms.4882)
Supplement: Supplementary file 2 — Figure S2. Identification of lipid molecules with major differences in spatial distribution. (A) Loading spectra for the first three principal components for the positive and negative DDA‐images in Figure 4A. (B) List of lipid molecules with most pronounced differences in topology. [file JMS-57-e4882-s002.pdf]

A

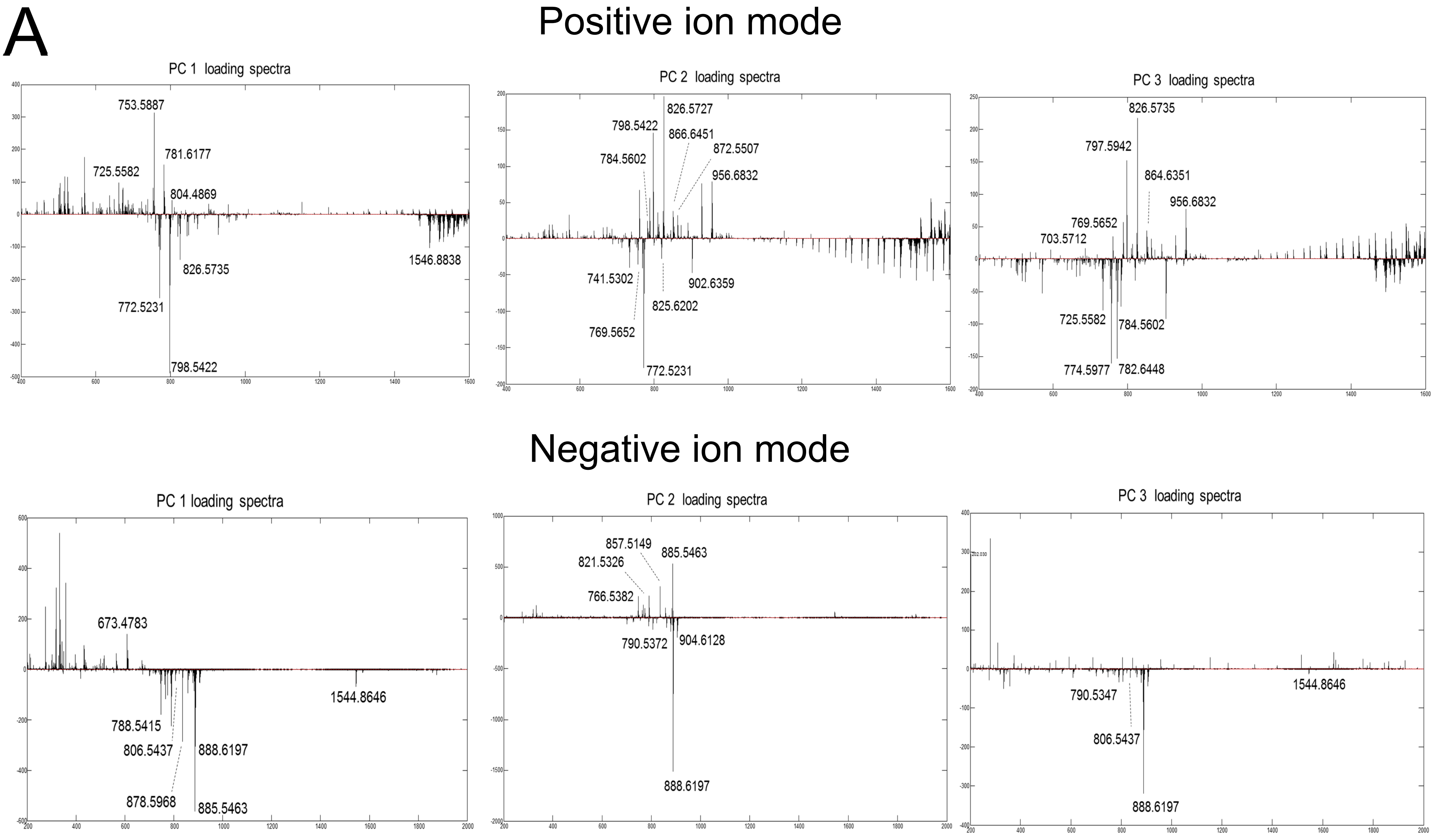

B

Lipid class	Lipid species IDed by ALEX^{123}	Molecular lipid species IDed by ALEX^{123}	Lipid detected with adducts	Calculated *m/z* [M+[39]K]$^+$	Measured *m/z* [M+[39]K]$^+$	ppm error	Calculated *m/z* [M-H]$^-$	Measured *m/z* [M-H]$^-$	ppm error
PA	PA 34:1	PA 16:0-18:1	-H+				673.4813	673.4783	-2.9
PC	PC 34:1	PC 16:0-18:1	+H+, +Na+,+[39]K+	798.5410	798.5422	1.5			
PC	PC 36:1	PC 18:0-18:1	+H+, +Na+,+[39]K+	826.5723	826.5735	1.5			
PC	PC 40:6	PC 18:0-22:6	+H+, +Na+,+[39]K+	872.5566	872.5584	2.1			
PC	PC O-34:1	PC O-16:1/18:0	+H+, +Na+,+[39]K+	784.5617	784.5602	-1.9			
PE	PE O-40:7	PE O-18:1/22:6	+H+; -H+				774.5432	774.5423	-1.1
PE	PE 38:4	PE 18:0-20:4	+H+; -H+				766.5381	766.5362	-2.5
PE	PE 40:6	PE 18:1-22:5	+H+; -H+,+Na+,+[39]K+				790.5381	790.5352	-3.6
PG	PG 40:6	PG 18:0-22:5	-H+				821.5327	821.5336	1.1
PI	PI 36:4	PI 16:0-20:4	-H+				857.5175	857.5149	-3.0
PS	PS 36:1	PS 18:0-18:1	-H+				788.5436	788.5415	-2.6
SM	SM 34:1;2		+H+, +Na+,+[39]K+	741.5307	741.5302	-0.7			
SM	SM 36:1;2		+H+, +Na+,+[39]K+	769.5620	769.5652	4.2			
SM	SM 38:1;2		+H+, +Na+,+[39]K+	797.5933	797.5942	1.1			
SM	SM 40:1;2		+H+, +Na+,+[39]K+	825.6246	825.6202	-5.3			
HexCer	HexCer 42:1;3		+Na+,+[39]K+	866.6482	866.6451	-3.6			
HexCer	HexCer 42:2;3		+Na+,+[39]K+	864.6325	864.6351	3.0			
HexCer	HexCer 40:1;3		+Na+,+[39]K+	838.6169	838.6151	-2.1			
SHexCer	SHexCer 36:1;2	SHexCer 18:1;2/18:0	-H+				806.5447	806.5457	1.2
SHexCer	SHexCer 40:1;3	SHexCer 18:1;2/22:0;1	-H+				878.6022	878.6058	4.1
SHexCer	SHexCer 42:2;2	SHexCer 18:1;2/24:1	-H+				888.6229	888.6197	-3.6
GM1	GM1 36:1;2	GM1 18:1;2/18:0	-H+				1544.8682	1544.8646	-3.0
